# The Influence of Neurotic Disorders and Neurobiological Basis on Eating Behavior in a 21-Year-Old Patient: A Case Study—Diagnostic Difficulties: Is It PANDAS/PANS in Relation to an Elevated ASO Level and the Diagnosis of OCD?

**DOI:** 10.3390/jcm15207878

**Published:** 2026-10-13

**Authors:** Agnieszka Zygo, Kamila J. Regin, Aleksandra Purkiewicz

**Affiliations:** 1 Dietetics Student, Faculty of Health Science, Collegium Medicum, University of Warmia and Mazury in Olsztyn, 10-900 Olsztyn, Poland; agnieszko.zygo@student.uwm.edu.pl; 2 Department of Rheumatology, Orthopaedics and Rehabilitation, School of Medicine, Collegium Medicum, University of Warmia and Mazury in Olsztyn, 10-228 Olsztyn, Poland; kamila.regin@uwm.edu.pl; 3 Department of Commodity Science and Food Analysis, Faculty of Food Science, University of Warmia and Mazury in Olsztyn, 10-726 Olsztyn, Poland

**Keywords:** OCD, disordered eating behaviors, PANDAS/PANS, ASO, QEEG, emotional regulation, psychosocial trauma, clinical case

## Abstract

**Background:** Anxiety and obsessive–compulsive disorders can co-occur with disordered eating behaviors and difficulties in emotional regulation. This article presents the clinical case of a 21-year-old female student whose complex neuropsychiatric profile required the simultaneous consideration of psychological, psychosocial, and somatic factors. The aim of this case report was to examine the coexistence of psychopathological symptoms, eating disturbances, psychosocial experiences, and somatic findings, while considering the potential role of post-infectious factors. **Methods:** A retrospective analysis of the patient’s medical and psychiatric history was conducted, including childhood infections, psychiatric diagnoses, psychosocial experiences, eating behaviors, and available laboratory findings. The assessment also included quantitative electroencephalography (QEEG) brain mapping performed under eyes-open and eyes-closed conditions. Particular attention was given to the retrospective hypothesis of PANDAS/PANS (Pediatric Autoimmune Neuropsychiatric Disorders Associated with Streptococcal Infections/Pediatric Acute-onset Neuropsychiatric Syndrome), while distinguishing documented clinical findings from unconfirmed etiopathogenetic hypotheses. **Results:** Retrospective history revealed recurrent upper respiratory tract infections and bilateral pneumonia in early childhood, followed by increasing anxiety and social withdrawal from peers. The patient was diagnosed with obsessive–compulsive disorder (OCD) at the age of 13, while psychiatric records from 2023 also documented depressive disorders and social phobia. Within the family system, the patient experienced relational trauma, including systematic verbal and psychological abuse by her father. Blood tests performed on 11 June 2025 revealed an elevated antistreptolysin O (ASO) level of 436 IU/mL, which may indicate previous streptococcal exposure but does not confirm active infection, PANDAS/PANS, or a causal relationship with neuropsychiatric symptoms. The available documentation lacked microbiological confirmation of streptococcal infections or evidence substantiating a PANDAS/PANS diagnosis. QEEG brain mapping revealed elevated fast HiBeta activity in the frontal regions under both recording conditions; however, incomplete methodological documentation precluded definitive interpretation of this finding as evidence of persistent cortical overactivity or specific cognitive deficits. Somatic and metabolic findings included morning cortisol of 7.7 µg/dL, dehydroepiandrosterone sulfate (DHEA-SO4) of 163 µg/dL, prolactin of 513.0 mIU/L, and total cholesterol of 138 mg/dL. A previously recorded glycemic value of 45 mg/dL was also noted, although insufficient information was available to confirm reactive hypoglycemia. The patient reported a dichotomous eating pattern characterized by appetite suppression during acute stress, followed by episodes of increased food intake aimed at regulating emotional tension. However, the absence of standardized assessment prevented confirmation of formal binge-eating episodes. Food avoidance and restriction were associated with contamination fears and obsessive–compulsive rituals. The patient also expressed marked body image dissatisfaction and a desire for an extremely thin physique, although no formal diagnosis of body dysmorphic disorder was documented. **Conclusions:** This case highlights the complex coexistence of obsessive–compulsive and anxiety symptoms, disordered eating behaviors, psychosocial trauma, and somatic findings. Although retrospective clinical history and elevated ASO levels raise the possibility of a post-infectious contribution, the available evidence does not establish an autoimmune mechanism or confirm PANDAS/PANS. The findings emphasize the importance of distinguishing documented clinical observations from etiopathogenetic hypotheses and support an interdisciplinary approach integrating psychiatric care, psychotherapy, nutritional assessment, and appropriate somatic diagnostics.

## 1. Introduction

Anxiety, obsessive–compulsive, and stress-related disorders, traditionally grouped under the umbrella term of neurotic disorders in the clinical literature, represent some of the most frequently diagnosed mental health conditions in the general population [1,2]. In modern diagnostic frameworks—the International Classification of Diseases (ICD-11) and the Diagnostic and Statistical Manual of Mental Disorders (DSM-5-TR)—the classic term ‘neurosis’ has been replaced by more precise categories, such as generalized anxiety disorder (GAD), panic disorder (PD), specific and social phobias, and obsessive–compulsive disorder (OCD) [3]. Despite these terminological updates, the historical term ‘neurosis’ continues to be utilized as a collective description of chronic emotional disturbances of multifactorial etiology, where anxiety, chronic tension, and preserved insight play a central role. The patient is typically aware of the irrationality of their experience, which further exacerbates psychological distress and impairs adaptive social functioning [4].

From a neurobiological standpoint, these disorders are underpinned by dysfunctions in the gamma-aminobutyric acid (GABA) and dopamine systems, amygdala hyperactivity, prefrontal cortex hypoactivation, and chronic activation of the hypothalamus–pituitary–adrenal (HPA) axis [5,6,7]. Chronic stress stimulates excessive cortisol secretion, which over the long term results in low-grade systemic inflammation, glucose metabolism dysregulation, insulin resistance, and a predisposition toward visceral adiposity [8,9]. This complex interaction highlights the bidirectional nature of the gut–brain axis, where psychological and emotional states directly modulate gastrointestinal motility, intestinal barrier permeability, and microbiota composition, while the gut and its microbiome reciprocally influence neurotransmitter synthesis, systemic inflammation, and anxiety levels [10,11].

In clinical dietetics, the correlation between neurotic disorders and eating behaviors is of fundamental importance. On one hand, anxiety and emotional tension trigger ‘emotional eating’, an attempt to alleviate negative effects by activating the brain’s reward system through foods rich in simple sugars and saturated fats [12]. On the other hand, acute stress in many patients induces painful spasms of gastrointestinal smooth muscle, total appetite suppression, and dyspeptic symptoms due to sympathetic dominance [13,14]. Consequently, individuals with neurosis frequently exhibit dichotomous eating patterns (cycles of restriction followed by compensatory binge eating), which foster deep micronutrient deficiencies (e.g., magnesium, ferritin, B-complex vitamins, zinc, omega-3 fatty acids) necessary for proper neuromodulation and neurotransmitter synthesis [15,16].

Modern therapeutic approaches in the nutritional prevention and management of neuroses emphasize the implementation of anti-inflammatory and neuroprotective dietary models, such as the Mediterranean Diet (MD) and the MIND (Mediterranean-DASH Intervention for Neurodegenerative Delay) diet [17]. These regimens are rich in polyphenols, antioxidants, and polyunsaturated fatty acids that stabilize neuronal function, support synaptic plasticity, and reduce anxiety [18,19]. The role of the clinical dietitian extends far beyond basic caloric balancing—it encompasses rebuilding a healthy relationship with food, psychoeducation on the impact of glycemia on mood, and close collaboration within an interdisciplinary team with psychiatrists and psychotherapists [20,21].

## 2. Case Presentation

This article aims to present the context illustrating the impact of neurotic disorders—including OCD—and somatic disorders on eating patterns. It describes the case of a 21-year-old female patient presenting with neuropsychiatric symptoms triggered by a streptococcal infection (a condition involving an autoimmune mechanism). The clinical picture included symptoms associated with food avoidance and restriction, alongside episodes of disinhibited eating behavior.

### 2.1. Clinical History and Medical Records

The present case is subject to limitations arising from the retrospective nature of the clinical history, the lack of complete medical records from childhood, the absence of data regarding family history and standardized psychiatric scale results, and the availability of only a single set of laboratory test results and incomplete information on quantitative electroencephalography (QEEG) methodology. The patient did not possess prior medical records. Furthermore, there is no formal record of the cognitive-behavioral therapy or the number of sessions the patient attended; the only available information comes from the patient herself, who stated that the therapy did not yield the results she had expected.

The case described concerns a 21-year-old female student who first exhibited emotional dysfunctions—including severe anxiety, social withdrawal, and difficulties adapting to her peer group—between the ages of 4 and 5. The onset of these symptoms coincided with a severe illness: bilateral pneumonia, followed by a series of chronic, recurrent upper respiratory tract infections. According to the patient, she also experienced infections during this period that she recalled as being bacterial; however, available medical records lack microbiological results confirming a streptococcal etiology. The patient also reports having been verbally informed by a physician that she had suffered from PANDAS (Pediatric Autoimmune Neuropsychiatric Disorders Associated with Streptococcal Infections) in childhood. Yet, there is no documentation to establish who made this diagnosis, when it was made, or on what criteria it was based. Consequently, in this article, PANDAS is presented as a retrospective diagnostic hypothesis rather than a confirmed diagnosis.

**Figure 1:**
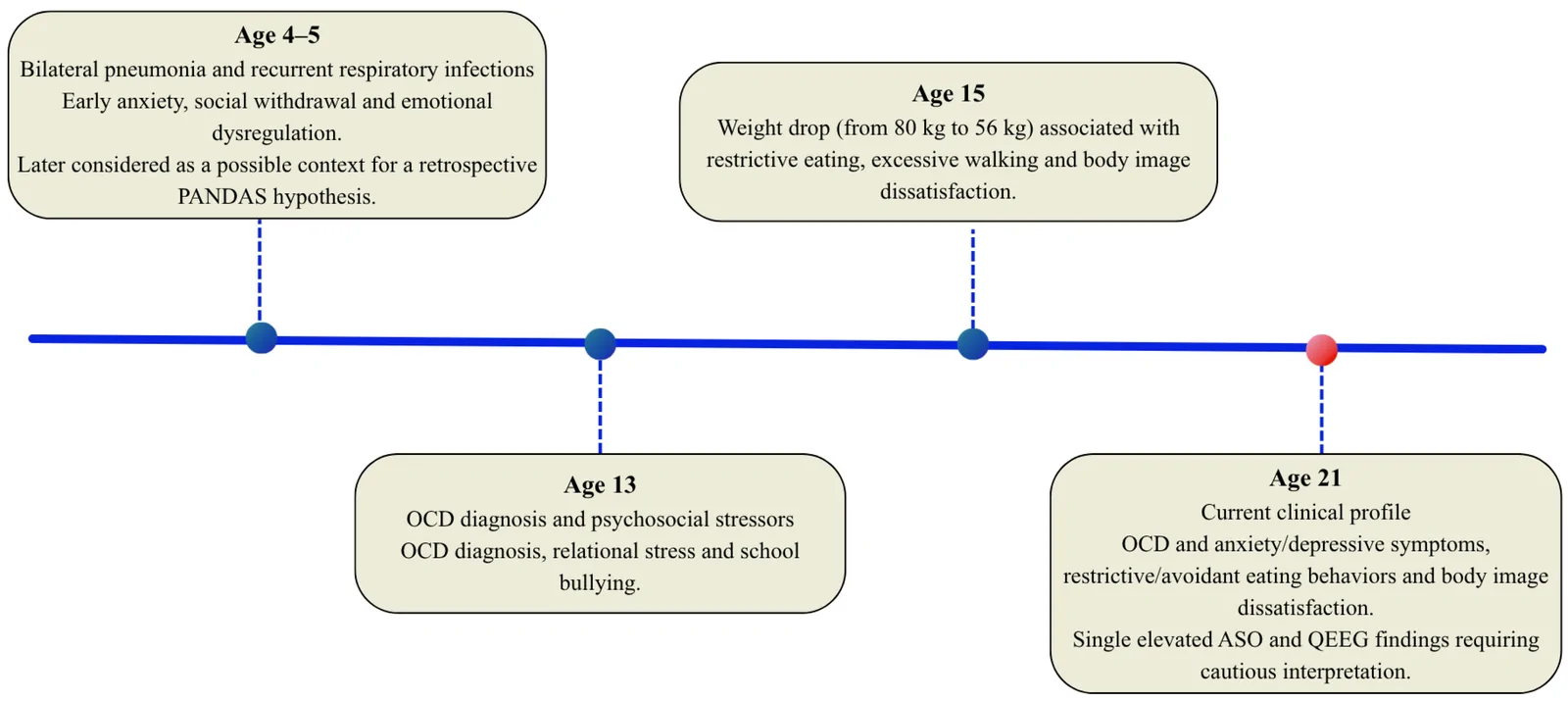
Chronological timeline of clinical and somatic psychopathology. Abbreviations: PANDAS—Pediatric Autoimmune Neuropsychiatric Disorders Associated with Streptococcal Infections; OCD—obsessive–compulsive disorder; ASO—antistreptolysin O; QEEG—quantitative electroencephalography.

During adolescence (at age 13), there was a drastic escalation of psychopathology, resulting in a formal diagnosis of OCD. Biological vulnerability was compounded by severe environmental stress, relational trauma within the family, and peer victimization. The patient was subjected to systematic bullying at school and constant verbal abuse from her father, who targeted her appearance and body weight; at the time, she was struggling with childhood obesity, weighing 80 kg at a height of 174 cm (Body Mass Index (BMI) 26.42 kg/m^2^). To cope with this traumatic rejection, she developed extreme compensatory behaviors: rigorous physical training, exhausting daily walks lasting several hours, and prolonged fasting. This led to a rapid, uncontrolled weight loss, dropping to 56 kg by the age of 15. The patient developed a distorted body image, which resulted in intense dissatisfaction with her appearance and reinforced a dichotomous cycle of eating behaviors that persist to this day. The chronological timeline of clinical and somatic psychopathology in the present case is presented in Figure 1.

### 2.2. Neurophysiological Profile and QEEG Assessment

To further the neuropsychiatric assessment, QEEG brain mapping was performed on the patient in 2021 under both eyes-open and eyes-closed conditions [22,23]. The patient had access only to the final summary of the QEEG report, which characterized a critically elevated percentage of high-frequency beta waves—associated with anxiety, stress, and racing thoughts (HiBeta waves)—in the frontal regions of the cerebral cortex, reaching 56% of relative power. This state indicates persistent cortical overactivity, hyper-reactivity, and a severely limited capacity for the body to enter a state of physiological rest and regeneration. Clinically, this manifests as an apathetic attitude, paralyzing anxiety regarding the initiation of actions, and a deeply ingrained negative cognitive bias. Due to the absence of full methodological documentation for the QEEG procedure, this result should be treated merely as a description contained within the report, without drawing definitive conclusions regarding permanent cortical overactivity or the underlying causes of the clinical symptoms.

Additionally, the report noted excessive arousal along the cortical midline. This finding was interpreted as being associated with brain regions involved in emotional regulation, impulse control, and cognitive flexibility. However, the lack of information regarding the full electrode montage, source localization procedures, and the methodology employed makes it impossible to attribute the observed recording to a specific deep structure, such as the anterior cingulate gyrus. The report also included a description of the dominant alpha frequency and the somatic symptoms reported by the patient. Due to the absence of complete methodological data and the lack of an independent neuropsychological assessment, however, one cannot draw conclusions regarding preserved semantic memory or attribute specific somatic symptoms to QEEG patterns based on this information.

Muscle artifacts (48–60 Hz) were recorded in the F7/F8 and T5/T6 regions during the examination; these may influence the interpretation of the quantitative and qualitative data.

Somatic complaints and muscle tension were also noted in the patient’s history; however, based on the available data, it is not possible to determine their etiology or directly attribute them to the QEEG findings. During periods of heightened stress, the patient exhibits repetitive body-focused behaviors (scratching the face, picking at skin lesions), which may be linked to attempts to alleviate psychological tension.

### 2.3. Laboratory, Metabolic, and Endocrine Profile

The laboratory assessment was based on the results of a single blood test panel and a general stool analysis, presented in Table 1 and Table 2. The available documentation did not provide sufficient data to confirm reactive hypoglycemia. The patient reported experiencing a slower heart rate, which might indicate bradycardia; however, the documentation lacked objective heart rate measurements during the described episodes. She also reported reduced vitality, difficulty concentrating, and sudden physical exhaustion necessitating daytime naps. The relationship between her symptoms, dietary habits, and glucose metabolism remains a hypothesis requiring further investigation. In particular, the absence of serial measurements of glucose, insulin, and C-peptide precludes confirmation of a reactive hypoglycemia mechanism.

The patient’s history also noted an episode where blood glucose measured 45 mg/dL. However, the available documentation provided no information regarding the measurement method, the timing of the test relative to meals, or the reproducibility of the result. Consequently, this result should not be cited as confirmation of reactive hypoglycemia.

A morning cortisol measurement showed a level of 7.7 µg/dL (reference range: 6.02–18.4 µg/dL), placing it within the laboratory’s stated reference range. A single morning measurement does not allow for the assessment of the diurnal cortisol profile or a diagnosis of “adrenal reserve exhaustion.” Dehydroepiandrosterone sulfate (DHEA-SO4) measured 163 µg/dL. As the available documentation does not provide a full reference range adjusted for age and sex, this result should not be interpreted as evidence of HPA axis dysfunction. A prolactin level of 513.0 mIU/L was also recorded, against a reference range of 102–496 mIU/L; this represents a slight elevation above the upper limit of the laboratory range. In the absence of a repeat measurement or data regarding sampling conditions, this single result cannot be definitively linked as a cause of the observed anxiety–depressive symptoms or eating behaviors. Total cholesterol was 138 mg/dL; this single result does not, in itself, constitute evidence of impaired steroidogenesis or metabolic insufficiency.

The biochemical panel showed a ferritin level of 27.4 ng/mL (reference range: 13.0–150.0 ng/mL), iron (Fe) at 89 µg/dL (range: 33–193 µg/dL), vitamin B12 at 337 pg/mL (range: 197–771 pg/mL), and folic acid at 6.06 ng/mL (range: 3.89–26.80 ng/mL). These values fell within the stated reference ranges; the ferritin level was at the lower end of the range. Based on these isolated results, neither deficiency nor malabsorption disorder can be diagnosed.

The eosinophil count was 0.54 × 10^3^/µL, representing 5.2% of the total leukocyte count. This percentage slightly exceeded the stated range of 0–5%, but the result alone does not indicate a parasitic infection. The erythrocyte sedimentation rate (ESR = 2 mm/h) was within the reference range of 0–12 mm/h. The gamma-glutamyl transferase (GGTP) value was 11 U/L (stated range: 12–43 U/L). A slight decrease in GGTP does not constitute evidence of liver failure or severe energy deficit.

The ASO result of 436.0 IU/mL from 11 June 2025 was elevated relative to the provided reference range. However, this is a single measurement and does not confirm an active streptococcal infection, a history of PANDAS, or a causal link between the infection and the neuropsychiatric symptoms. The documentation does not present serial ASO measurements or the results of cultures or other microbiological tests confirming a streptococcal etiology.

General stool analysis revealed brown, formed stools with a pH of 7.0, falling within the reference range of 7.0–8.0. No fat droplets, starch granules, or striated muscle fibers were observed in the sample. However, the results presented do not constitute a sufficient basis on their own to diagnose malabsorption syndrome or any other specific gastrointestinal pathology.

### 2.4. Mixed Presentation of Eating Disorders and OCD

The patient’s eating behaviors follow a two-phase pattern closely linked to emotional tension. In the first phase—triggered by an acute stressor—she describes a sensation of intense tightness in her abdomen and an inability to consume food. In the second phase—after the immediate stressor has subsided—she reports increased food intake as a means of regulating emotional tension. Her dietary choices are dominated by peanut butter, sweets, and chicken. For instance, she reports consuming the contents of an entire jar of peanut butter, a chocolate bar, other sweets, and chicken within the span of a few hours. Her preference for chicken stems from the fact that it is one of the very few foods that does not trigger a profound sense of disgust or a fear of contamination.

The patient has developed rigid rules associated with her OCD that directly impact her eating habits. These rules concern both qualitative aspects (hygiene protocols, requirements for fork and plate sterility, and a fear of touching surfaces with the fork) and quantitative ones (a strict energy limit of exactly 2000 kcal per day). Any deviation from these rules or exceeding the limit triggers intense anxiety. Following an episode of increased food intake, the patient reports employing dietary restrictions and fasting in the subsequent days to neutralize her anxiety and regain a sense of control. These behaviors may serve to regulate anxiety and restore a sense of control; however, their precise psychological function would require direct clinical assessment.

As of June 2025, her anthropometric status was 64 kg in weight and 179.5 cm in height, corresponding to a BMI of 19.9 kg/m^2^. The patient described this body weight as highly unsatisfactory and unacceptable. She reported intense dissatisfaction with her appearance and a desire for a very slender physique. However, the available documentation does not present a formal diagnosis of body dysmorphic disorder (BDD); therefore, this diagnosis should not be considered confirmed.

Due to the lack of standardized assessment regarding the frequency, duration, and characteristics of episodes of increased food intake, the available data do not allow for a determination of whether these episodes meet the full diagnostic criteria for a binge-eating episode (BED). Similarly, while the description of avoidant and restrictive behaviors may resemble certain features of avoidant/restrictive food intake disorder (ARFID), the absence of a formal diagnosis precludes confirmation of this condition.

### 2.5. Past Pharmacotherapy, Supplementation, and Interventions

Previous pharmacological treatment included, among others, Lambotrix (lamotrigine), Xetanor (paroxetine hydrochloride), Spamilan (buspirone hydrochloride), and Xanax (alprazolam). According to the patient, the treatment did not yield the expected results; however, she could not recall the dosages taken or the full duration of the individual therapies. Consequently, based on the available data, it is impossible to assess the degree of response to specific medications or to identify drug resistance. Furthermore, there are no grounds to attribute the ineffectiveness of the pharmacotherapy to the QEEG profile.

Currently, the patient is taking Faxolet ER (venlafaxine)—a serotonin-norepinephrine reuptake inhibitor—at a dose of 150 mg, as well as Izotek (isotretinoin) for acne. She also used supplements including N-acetylcysteine, lactoferrin, zinc, B vitamins, magnesium, Omega-3 fatty acids, potassium iodide, and digestive enzymes intended to improve post-meal comfort. Biofeedback training was also recommended. However, the available documentation does not present a standardized assessment of the efficacy of these interventions or systematic monitoring following their use.

## 3. Discussion

### 3.1. PANDAS/PANS as a Retrospective Diagnostic Hypothesis

The etiology of the patient’s disorder is multifactorial. Between the ages of 4 and 5, the patient suffered from severe bilateral pneumonia; according to her retrospective account, this was followed by recurrent upper respiratory tract infections, as well as escalating anxiety, social withdrawal, and adjustment difficulties. While the severe pneumonia can be viewed as a significant biological event and a potential early-life stressor, the temporal coincidence between it and the onset of symptoms does not allow for the establishment of a direct causal link to the subsequent development of OCD, nor does it confirm a post-infectious mechanism. This chronology provides grounds for considering a retrospective PANDAS/PANS hypothesis, yet it is insufficient to confirm such a diagnosis. The available documentation contains no data indicating who diagnosed the patient with PANDAS, when the diagnosis was made, or what criteria were used. The patient reports only having been informed verbally of such a diagnosis during childhood. She also lacks complete medical records from that period and any microbiological test results confirming a streptococcal infection.

The term PANDAS (Pediatric Autoimmune Neuropsychiatric Disorders Associated with Streptococcal infections) was introduced to describe a subgroup of children in whom symptoms of OCD or tic disorders occur in temporal association with Group A streptococcal infections [24]. Proposed pathophysiological mechanisms have highlighted, among other factors, the possibility of molecular mimicry between streptococcal antigens and neuronal structures, as well as the involvement of immunological mechanisms. This hypothesis was partly inspired by clinical similarities to Sydenham’s chorea; however, the autoimmune mechanism underlying PANDAS has not been fully confirmed [25].

In the case presented, the interpretation of the ASO result is of particular importance. On 11 June 2025, the patient was found to have an elevated ASO level of 436 IU/mL. This is a single measurement and does not allow for the confirmation of an active streptococcal infection, a history of PANDAS/PANS, or a causal link between the infection and the neuropsychiatric symptoms. The available documentation contains no serial ASO measurements, throat culture results, or other microbiological test findings documenting recurrent streptococcal infections.

Cases of adults with similar symptoms following streptococcal infections have been described in the literature [26,27]. However, these reports concern different patients and do not constitute evidence for a PANDAS diagnosis in the patient presented here. Similarly, histopathological and population-based reports regarding potential immunological mechanisms of PANDAS/PANS should be viewed as the background literature rather than evidence of such changes in the patient described. No brain tissue analysis was performed in this case; therefore, there is no evidence confirming the presence of inflammation, immune cell infiltration, or an autoimmune reaction within the basal ganglia. There is insufficient data to confirm that the patient met the full criteria for PANDAS/PANS, specifically regarding the sudden onset of symptoms, documentation of a streptococcal infection, and the precise chronological link between the infection and the onset or exacerbation of OCD. Consequently, PANDAS/PANS should be regarded as a diagnostic hypothesis requiring further verification, rather than a confirmed etiology of the disorder.

### 3.2. Diagnostic Criteria for PANDAS/PANS and Their Limitations

Proposed criteria for PANDAS include, among others, symptom onset during childhood, the sudden appearance or significant exacerbation of OCD or tic disorders, and a temporal association with streptococcal infection [24]. For PANS, the criteria have been expanded to include the sudden onset of OCD or severely restricted food intake, alongside the concurrent presence of at least two additional categories of neuropsychiatric symptoms [28]. The broad definition of PANS necessitates particularly careful differential diagnosis against other neurological, psychiatric, and general medical conditions. While clinical criteria can assist in identifying patients who require further diagnostic evaluation, they should not be regarded as standalone evidence of an autoimmune mechanism underlying the disorders [29].

In the present case, there are certain elements consistent with the post-infectious syndrome hypothesis, primarily the early onset of symptoms and a retrospectively reported temporal association with infectious illnesses. At the same time, the lack of complete pediatric records, the absence of microbiological confirmation of streptococcal infections, and the lack of documentation regarding the initial PANDAS diagnosis significantly limit the feasibility of applying these criteria retrospectively.

### 3.3. Neuroimmunological Mechanisms of OCD and the Secondary OCD Hypothesis

OCD is a disorder with a complex etiopathogenesis involving the interplay of genetic, neurobiological, psychological, and environmental factors [30]. Neurobiological studies attach particular importance to abnormalities within cortico–striato–thalamo–cortical (CSTC) circuits [31,32], as well as alterations in serotonergic, dopaminergic, and glutamatergic neurotransmission [33,34].

In recent years, the potential involvement of immunological and inflammatory mechanisms in a subset of OCD cases has also been investigated [33,35,36]. While some studies have reported differences in cytokine levels or the presence of antibodies, the findings remain inconclusive. For instance, a meta-analysis of serum cytokine levels found no significant differences between individuals with OCD and a control group [37]. Consequently, the possible role of immunological mechanisms remains a subject of ongoing research and cannot be automatically attributed to every patient with OCD.

The issue of so-called secondary OCD is significant for differential diagnosis, particularly when symptoms emerge in the context of specific neurological, autoimmune, or metabolic disorders [38]. In the case presented here, there is insufficient evidence to diagnose secondary OCD of autoimmune origin. Although a retrospective post-infectious hypothesis might be considered, available data do not support the conclusion that the OCD symptoms stem from neuroinflammation or autoimmune damage to brain structures involved in behavioral regulation. Regarding the treatment of PANDAS/PANS and suspected secondary forms of OCD, the literature describes various therapeutic strategies; these include standard OCD treatments and, in selected clinical situations, interventions targeting the potential infectious or immunological trigger [39,40]. Given the absence of a confirmed PANDAS/PANS diagnosis in the patient described, this information should be regarded as background from the literature rather than a basis for administering specific treatment to this individual.

It is also worth noting that non-invasive neuromodulation methods—including transcranial magnetic stimulation (TMS) and transcranial direct current stimulation (tDCS)—have been investigated in recent years [41]. These methods have been studied in the context of conditions such as obsessive–compulsive disorder, as well as specific eating and emotion regulation disorders [42,43,44]. Available findings suggest a potential reduction in the severity of certain symptoms; however, efficacy depends on the specific method and protocol employed, and the data remain inconclusive. Regarding the patient in question, there is insufficient evidence to support the use of TMS or tDCS as a specific treatment for her clinical profile.

### 3.4. OCD and Disordered Eating Behaviors

Eating disorders (EDs) represent another area contributing to the multidimensional nature of a patient’s suffering and the presence of overlapping diagnoses. According to the literature, EDs exhibit higher rates of comorbidity with OCD than are observed in the general population [45]. Epidemiological analyses indicate that the rate of this comorbidity is similar among patients with anorexia nervosa (AN), 35–44%, and bulimia nervosa (BN), ranging from 35% to 40%. In the case of BED, the rate of co-occurrence with OCD hovers around 2.2% (with a maximum of 8.2%), a figure that falls within the range of the standard lifetime prevalence for this disorder [46].

Among young people with ARFID, OCD co-occurs in approximately 4% of cases [47]. However, there is a lack of data regarding the prevalence of OCD co-occurring with other specified eating disorders, highlighting the need for further research in this area. The literature reports varying prevalence rates of OCD among individuals with eating disorders, ranging from 0% to 59% for AN, 3% to 30.1% for BN, and 0% to 8.2% for BED [48].

OCD frequently co-occurs with AN, yet the mechanism underlying this relationship remains not fully understood [49]. Proposed explanations point to a shared genetic and neurobiological basis, linked—among other factors—to cognitive rigidity and an intense need for control [50]. Alternatively, it has been suggested that OCD symptoms may develop secondary to AN or that both disorders partially share common psychopathological mechanisms [51]. The differential diagnosis of AN and OCD requires consideration of other mental disorders and somatic conditions that may present similar symptoms. In the case of OCD, it is necessary to differentiate the condition from, among others, anxiety disorders, eating disorders, depression, body dysmorphic disorder, ASD, substance use disorders, psychoses, and tic disorders [52,53]. Conversely, the diagnosis of AN necessitates ruling out somatic diseases and mental disorders that could lead to weight loss and restricted food intake [54]. In the case described, the initial diagnosis of AN was based on the presence of significant weight deficit, a distorted body image, and restricted food intake. However, during continued observation, intensified obsessive–compulsive symptoms were noted, including hygiene rituals lasting several hours, repetitive behaviors, perfectionism, and excessive scrupulosity. The symptoms of OCD and AN emerged around the same time, making it difficult to clearly distinguish between the two disorders. This case highlights the importance of thorough differential diagnosis and consideration of the potential co-occurrence of OCD and AN [55].

### 3.5. Gut–Brain Axis

**Figure 2:**
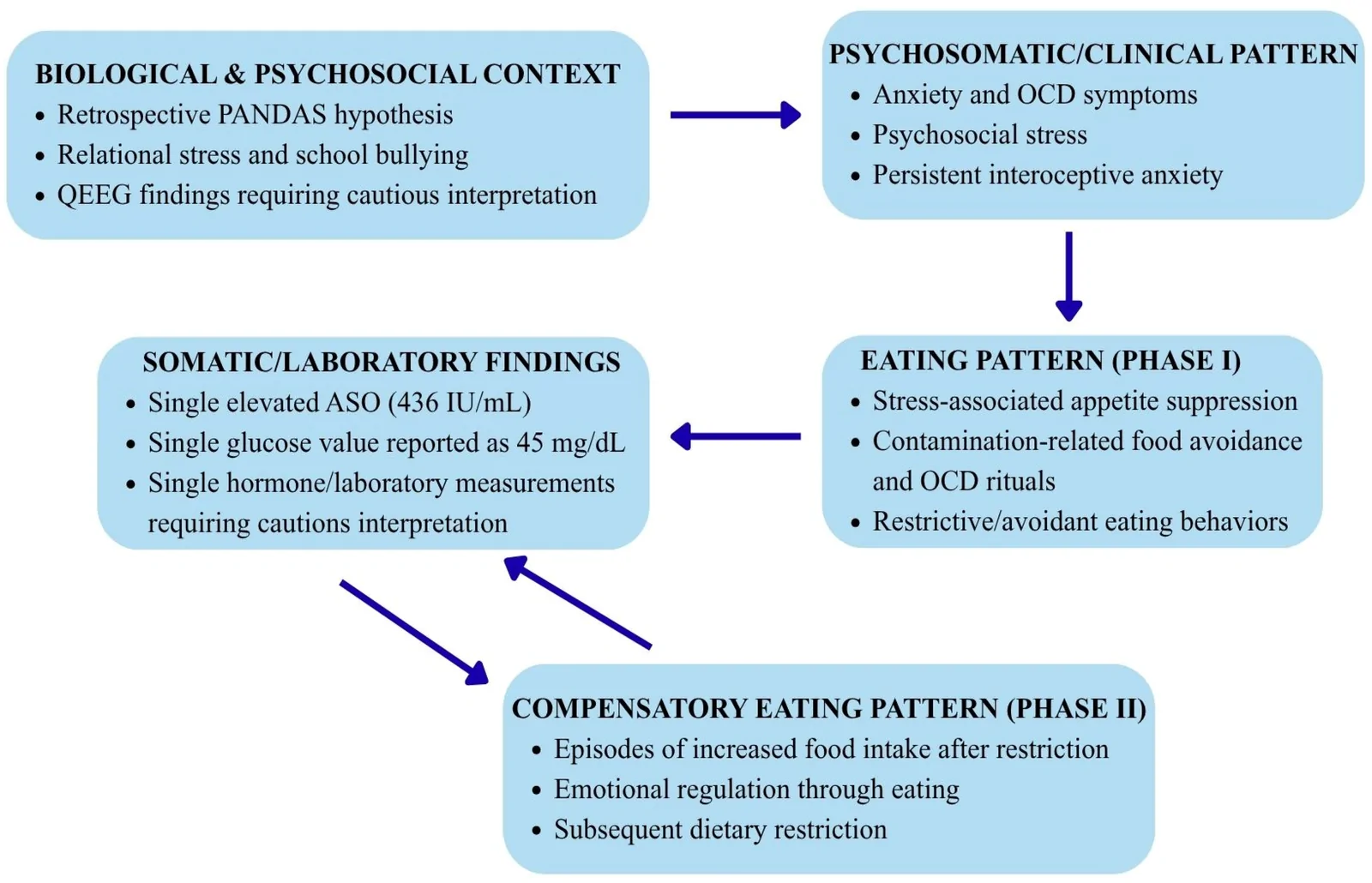
Pathophysiological Feedback Loop of the Gut–Brain Axis & Metabolic Instability. Abbreviations: PANDAS—Pediatric Autoimmune Neuropsychiatric Disorders Associated with Streptococcal Infections; QEEG—quantitative electroencephalography; OCD—obsessive–compulsive disorder; ASO—antistreptolysin O.

The gut–brain axis is a bidirectional communication system between the gastrointestinal tract and the central nervous system, involving neuronal, hormonal, metabolic, and immunological mechanisms, among others [10]. The literature suggests possible links between alterations in gut microbiota, microbial metabolites, and emotional and neuropsychiatric functioning [56]. Similar associations have also been analyzed in the context of eating disorders [57,58]. However, in the case of the patient described here, neither microbiome analysis nor tests enabling the diagnosis of dysbiosis or the identification of changes within the gut–brain axis were performed. Consequently, its potential role in clinical presentation should be regarded as a hypothesis rather than a confirmed mechanism. The feedback loop illustrated in Figure 2 provides a conceptual framework for considering the potential ninterplay between gastrointestinal, metabolic, and neuropsychiatric symptoms in the presented case.

The possibility that the psychotropic medications used may have influenced gastrointestinal function or the gut microbiota must also be considered. Available studies suggest that antidepressants can affect microbiome composition, although the clinical significance of these changes remains inconclusive [59,60]. In the case of the patient described, there is no microbiological data available to assess such an impact; therefore, the potential role of pharmacotherapy in this regard remains hypothetical.

### 3.6. Psychosocial Factors, Trauma, and Eating Behaviors

A significant element of the patient’s history involves experiences of verbal and psychological abuse within the family environment, as well as peer victimization. At age 13—a period marked by heightened psychosocial stress—she was diagnosed with OCD; subsequently, she engaged in restrictive eating behaviors, intense exercise, and fasting, leading to a weight drop to 56 kg by age 15.

The literature indicates that traumatic experiences and chronic stress can co-occur with the intensification of eating disorder symptoms, difficulties in emotional regulation, a negative body image, and a need to regain control [61,62]. Studies on adolescents with eating disorders also point to links between the severity of traumatic experiences and certain bulimic symptoms and compensatory behaviors [63,64].

In the case of the patient presented here, experiences of abuse and chronic stress should be regarded as significant psychosocial factors co-occurring with the progression of symptoms, rather than as a directly proven cause of body image disturbances or eating behaviors. Such a cautious interpretation allows for the acknowledgment of the significance of environmental experiences without attributing an undocumented causal role to them.

From a clinical perspective, the interplay between anxiety symptoms, OCD, psychosocial experiences, and eating behaviors may be of particular importance. At the same time, it should be noted that the case report does not make it possible to determine whether any of these factors were the primary cause of the other symptoms.

### 3.7. Clinical Significance of the Presented Case

The case presented highlights the need for a comprehensive assessment of patients presenting with co-occurring obsessive–compulsive symptoms, disordered eating behaviors, chronic stress, and nonspecific laboratory abnormalities.

Caution is required when interpreting isolated laboratory results as evidence of specific pathophysiological mechanisms. In this case, a single ASO titer, a solitary cortisol measurement, a slight prolactin deviation, a GGTP result, and a recorded blood glucose level of 45 mg/dL are insufficient to independently confirm a specific etiology for the neuropsychiatric disorders or to establish a direct mechanism underlying the eating behaviors.

Similarly, the QEEG result must be interpreted with due regard for methodological limitations. Only the final report was available, lacking full data regarding the montage, acquisition parameters, data processing, source localization, and the normative database used. Consequently, the QEEG result cannot be used to confirm a specific neuroanatomical dysfunction or to explain the efficacy—or lack thereof—of pharmacotherapy.

Overall, this case illustrates the importance of an interdisciplinary approach encompassing psychiatric, psychotherapeutic, nutritional, and somatic assessments. At the same time, the data presented should be interpreted as a basis for formulating diagnostic and clinical hypotheses rather than as proof of specific cause-and-effect relationships. The presented case analysis and the review of the scientific literature highlight the complex, multidimensional, and interactive etiopathogenesis of the neuropsychiatric symptoms observed in the patient. The clinical presentation demonstrates a clear overlap of three main etiopathogenetic domains:

1. Post-infectious immunological component (PANDAS/PANS/secondary OCD): Indications point to a history of recurrent streptococcal infections in childhood, bilateral pneumonia, and elevated ASO titers, which may have triggered autoimmune processes (molecular mimicry, neuroinflammation involving reactive astrogliosis and T-cell responses within the basal ganglia).
2. Psychotraumatological factors: Adolescent trauma (domestic violence, a hostile environment, stigmatization) acting as a direct trigger for drastic compensatory behaviors, deficits in affective self-regulation, and body image disturbances.
3. OCD–ED comorbidity: An overlap of cognitive rigidity, perfectionism, a need for control, and intropunitive behavioral patterns, leading to a redefinition of the initial diagnosis of AN toward a manifestation of obsessive–compulsive disorder (OCD) with accompanying disordered eating.

This case highlights the hypothesis that, in some patients initially classified as having treatment-resistant forms of OCD or ED, the underlying cause of symptoms is secondary—driven by neuroinflammatory and post-infectious processes superimposed on psychological vulnerability and environmental trauma.

### 3.8. Limitations of the Study

The interpretation of the presented case is limited by the retrospective nature of the collected history, incomplete childhood medical records, the absence of data regarding family history and standardized psychiatric assessment results, and incomplete methodological documentation of the QEEG study. The lack of childhood records makes it impossible to verify who diagnosed PANDAS, when it was diagnosed, or the clinical grounds for the diagnosis, nor does it allow for determining whether documented streptococcal infections occurred during that period. The available ASO titer result from 11 June 2025 represents a single measurement and cannot be interpreted as confirming a PANDAS diagnosis or as evidence of an active streptococcal infection.

Regarding the QEEG study, only the result description was available, lacking full information on the electrode montage used, signal acquisition parameters, frequency band definitions, artifact identification and removal procedures, source localization methods, and the normative database applied. Consequently, the QEEG results should be viewed as part of the case description and study report rather than as an independent basis for inferring a specific neuroanatomical or neurophysiological dysfunction.

An additional limitation is the lack of available data concerning the parents’ psychiatric history and prenatal or perinatal factors, including potential medication exposure during pregnancy. Incomplete documentation also makes it impossible to determine whether the patient had previously undergone cognitive-behavioral therapy (CBT). The lack of information regarding any prior use of CBT, including its scope, duration, intensity, and the clinical response, limits the ability to evaluate past psychotherapeutic interventions and their potential impact on the course of symptoms.

The absence of systematic, prospective monitoring following the interventions implemented or proposed further limits the ability to reliably assess their efficacy and the durability of the results achieved. Consequently, the case presented should be interpreted as a description of a complex clinical picture, along with associated diagnostic and etiopathogenetic hypotheses that require further verification; it does not provide a basis for drawing definitive conclusions regarding cause-and-effect relationships between specific factors and the observed symptoms.

## 4. Conclusions

The case presented here is subject to limitations arising from the retrospective nature of clinical history, the absence of family history, incomplete medical records and psychiatric assessment data, the availability of only isolated laboratory test results, and a lack of full information regarding the QEEG methodology employed. The patient did not possess prior medical records. This case demonstrates that disordered eating behaviors may stem from a complex interplay of psychological, neurobiological, and somatic factors. Key elements of the clinical presentation included eating rituals, a need to control caloric intake and body weight, and the intense avoidance of specific foods. The potential role of reactive hypoglycemia remains a hypothesis, as the available data do not allow for its confirmation. Traumatic factors and emotional dysregulation also played a significant role, potentially influencing the patient’s relationship with food and her own body. Consequently, comprehensive diagnosis and treatment require an interdisciplinary approach encompassing psychotherapy, psychiatric care, nutritional therapy, and somatic assessment. Considering the interconnections between these areas is crucial for the effective planning of therapeutic management.

## Figures and Tables

**Table 1:** Detailed Summary of the Patient’s Laboratory and Endocrine Profile.

Parameter Assessed	Patient’s Result	Reference Range
Antistreptolysin O (ASO)	436.0 IU/mL **↑**	0.0–200.0 IU/mL
Fasting Glucose	82 mg/dL **↔**	70–99 mg/dL
Prolactin	513.0 mIU/L **↑**	102–496 mIU/L
Dehydroepiandrosterone sulfate (DHEA-SO4)	163.0 µg/dL ↔	Age norm: ~320 µg/dL
Morning Cortisol	7.7 µg/dL ↔	6.02–18.4 µg/dL
Total Cholesterol	138 mg/dL ↔	Recommended: >190 mg/dL
Ferritin	27.4 ng/mL ↔	13.0–150.0 ng/mL
Vitamin B12/Folate	337 pg/mL ↔	197–771 pg/mL
Gamma-glutamyl transferase (GGTP)	11 U/L **↓**	12–43 U/L
Eosinophils	5.2% **↑**	0–5%

Abbreviations: ↑ above reference range; ↔ within reference range; ↓ below reference range. Source: Own data collected as part of the case study.

**Table 2:** Results of Routine Stool Examination.

Parameter	Result
Color	Brown
Consistency	Compact
Odor	Characteristic
pH	7.0 (reference range: 7.0–8.0)
Fat droplets	Not detected in the specimen
Starch granules	Not detected in the specimen
Striated muscle fibers	Not detected in the specimen

Source: Own data collected as part of the case study.

## Data Availability

No new data were created in this study.

## Abbreviations

The following abbreviations are used in this manuscript:

ARFID	Avoidant/Restrictive Food Intake Disorder
BDD	body dysmorphic disorder
HPA	hypothalamus–pituitary–adrenal axis
OCD	obsessive–compulsive disorder
PANDAS/PANs	Pediatric Autoimmune Neuropsychiatric Disorders Associated with Streptococcal Infections Syndrome/Pediatric Acute-onset Neuropsychiatric Syndrome
QEEG	quantitative electroencephalography
